# Proton pump inhibitors as a risk factor for norovirus infection

**DOI:** 10.1017/S0950268817000528

**Published:** 2017-03-22

**Authors:** C. PRAG, M. PRAG, H. FREDLUND

**Affiliations:** 1 Department of Laboratory Medicine, Örebro University Hospital, Örebro, Sweden; 2 Department of Infectious Diseases, Örebro University Hospital, Örebro, Sweden; 3 Department of Laboratory Medicine, Örebro University Hospital, Faculty of Medicine and Health, Örebro University, Örebro, Sweden

**Keywords:** Norovirus infection, proton pump inhibitors, risk factor

## Abstract

Norovirus causes viral gastroenteritis, which is a major problem in health care. The disease causes death in elderly and seriously ill patients, and results in significant health costs each year. Proton pump inhibitors (PPIs) reduce gastric acidity, which is an important protection against microorganisms. We hypothesised that treatment with PPIs increases the risk of contracting norovirus infection. This has not previously been studied. The study was a retrospective case–control study, in which 192 hospitalised patients positive for norovirus in Örebro County, Sweden, were identified as cases. For each case, a hospitalised patient who did not have the infection was selected as a control, and matched with respect to ward, gender, admission date and age. Details of exposure, i.e. treatment with PPIs, were retrieved from the patient records. Odds ratio (OR) with confidence intervals (CIs) and *P*-values were calculated using McNemar's test. There was a significantly increased risk of norovirus infection in patients treated with PPIs compared with patients without PPI treatment (OR 1·73, 95% CI 1·07–2·81; *P* = 0·02). PPIs appear to be a risk factor for norovirus infection, and our results motivate future studies to further examine this association.

## INTRODUCTION

Viral gastroenteritis affects approximately one million Swedes each year, causing attacks of vomiting and diarrhoea [1]. The disease is often caused by human caliciviruses, specifically noroviruses and sapoviruses, with norovirus genogroup II being the most common type in humans [1, 2]. In in-patient care, viral gastroenteritis affects both patients and staff. The disease causes increased mortality in elderly and seriously ill patients and results in large economic losses due to sick leave and need to isolate infected patients [1].

It is important to identify patients at elevated risk for norovirus infection to prevent the spread of this disease. Gastric acid is an important factor in terms of defence against microorganisms. The daytime average gastric pH in humans is about 1·4 [3]. Proton pump inhibitors (PPIs) are drugs that are extensively used [4] as treatment for many conditions, such as dyspepsia, peptic ulcer and gastro-oesophageal reflux disease (GORD). These substances reduce the number of hydrogen ions in gastric juice by inhibiting parietal cell secretion of hydrogen ions, which increases the gastric pH [3]. Furthermore, PPIs affect the gut flora, leading to overgrowth of bacteria in the gastrointestinal (GI) tract [5]. The drugs have also been proved to reduce motility in the GI tract and, further, to reduce the effects of the immune system [6–9]. The therapeutic targets for PPIs are pH >3 (peptic ulcers) and >4 (GORD) [3].

Historically, it has not been possible to cultivate human norovirus *in vitro*, a problem that may now have a solution [10]. Previously, surrogate viruses have been used to investigate the stability at various pH levels [11]. In these studies, human norovirus is assumed to be stable at low pH; no studies have been conducted in which surrogates are exposed to pH <2. Murine norovirus is stable at pH 2, with a moderate titre reduction (1-log reduction after 2·5 h) compared with the reduction (4-log reduction after 2·5 h) in feline calicivirus [11]. However, comparing surrogates with human norovirus is complicated, as these viruses may differ in stability even though they are similar in other respects [2].

The hypothesis of this study is that PPI treatment increases the risk of contracting norovirus infection. There are, to the best of our knowledge, no previously published studies investigating this relationship.

## METHODS

### Study design

This was a retrospective case–control study. Cases were identified from the database of the Clinical Microbiological Laboratory, Örebro University Hospital, Örebro, Sweden. They were hospitalised patients who had tested positive for norovirus genogroup II by PCR during the period 1 January 2011–1 September 2014. Norovirus genogroup II is the most common cause of both norovirus outbreaks and sporadic cases of norovirus infection [12]. Each case was individually matched with a control patient, using a list of in-patients from the years 2011–2014. In order to avoid confounding, matching was done with respect to ward, gender, admission date (±1 month) and age (±5 years). Patients could only be included in the study once, either as case or as control. This was checked using the participants’ personal identity numbers. All three hospitals in Örebro County were included in the study, Örebro University Hospital, Karlskoga Hospital and Lindesberg Hospital.

### Laboratory testing

The norovirus diagnostic method used from 2011 to January 2014 was RNA-extraction with Bullet BUGS'n BEADS™, Bullet (DiaSorin, Dublin, Ireland) followed by an in-house real time-PCR using SuperScript™ III Platinum® One-Step Quantitative RT-PCR System (Invitrogen, Carlsbad, CA) on LightCycler 2·0, (Roche Diagnostics GmbH, Mannheim, Germany). The diagnostic method used since February 2014 is automatic PCR analyses using the Enteric Viral Panel (Diagenode, Seraing, Belgium & BD) with BD Max (Becton, Dickinson and Company, Dublin Ireland).

### Study population

Cases of norovirus infection were defined as adult patients with a positive test for norovirus genogroup II and symptoms of gastroenteritis as documented in the medical records. The symptoms were vomiting and/or diarrhoea, having no other explanation, either reported from the patient or observed by the medical staff. Norovirus genogroup II-positive patients with a discharge code indicating gastroenteritis were also defined as cases. Only patients who developed symptoms of gastroenteritis within the period of hospitalisation were regarded as cases.

Controls were defined as patients without any clinical suspicion of viral gastroenteritis documented in the medical records and they were subsequently not tested for norovirus infection. No control patient had a discharge code indicating gastroenteritis.

Inclusion criteria were firstly an admission record in which current medicines were reported, and secondly age ⩾18 years at the time of admission. Gastroenteritis was not suspected at admission in any of the study patients. Patients with a hospital stay of <2 days were excluded, since the incubation period for norovirus normally is about 24 h [13]. A 2-day hospitalisation was considered minimum to determine whether the patient had contracted the infection at the ward. The criteria was used to ensure that the exposure to the virus was similar for cases and controls. Patients on PPIs to be taken ‘as needed’, according to the admission records, were excluded as it was not possible to determine whether they were using PPIs at hospital admission. Inclusion criteria were met by 195 cases, three of whom were excluded because of lack of controls with matching criteria. This resulted in 192 case–control pairs.

### Data collection

Information on whether the patients were being treated with PPIs was retrieved from the electronic medical record system ‘Klinisk Portal’. Since PPIs are available both by prescription and over the counter, we retrieved information about PPI therapy from the admission records, instead of the patients’ electronic medication lists. In the admission records, current medications are documented based on information from the patient. PPI therapy was defined as daily use of PPIs, with substance names or trade names given as listed in *Farmaceutiska Specialiteter i Sverige (FASS)*.

### Statistical analysis

Estimation of the number of cases and controls needed to prove a significant correlation in the study was made with StatCalc in Epi Info 7·0 using Fleiss with continuity correction. Assuming that 20% of the controls were exposed at an odds ratio (OR) of 2, altogether 187 cases and 187 controls were required to achieve 80% power at 5% significance (two-sided). Since no previous studies in the field have been performed, the estimation of ORs was based on how PPIs increase the risk of infection with *Clostridium difficile* [14]. An OR of 2 was considered to indicate sufficient power to warrant intervention regarding PPIs.

Descriptive statistics were calculated in Excel 2016 version 15·26 using means, medians and standard deviations (s.d.). Pairwise statistical analysis was performed as the study participants were individually matched. Data were analysed with StatCalc in Epi Info 7·0 and its Pair-Matched Case–Control analysis method using a 2 × 2 table. An OR with 95% confidence intervals (CIs) and *P*-values was calculated according to McNemar's method. A *P*-value <0·05 was considered significant.

## RESULTS

### Study population

At the three hospitals in Örebro County, we studied 192 case–control pairs. Mean age was 79·8 years for cases and 79·6 years for controls; 51·6% of the patients were female. The majority (55·7%) had been admitted to Örebro University Hospital, 32·8% to Karlskoga Hospital and 11·5% to Lindesberg Hospital. Of the 23 departments surveyed, 15 were within Örebro University Hospital. The largest proportion of patients (78·1%) were treated in a medical/geriatric ward and only 6·3% were treated in a surgical ward. The mean stay in hospital was 16·7 days for cases and 10·9 days for controls. Hospital stay was a minimum of 2 days and a maximum of 82 days for the cases, and 2 days’ minimum and 52 days’ maximum for controls. The median length of hospitalisation stay was 12 days for cases and 7 days for controls, and the median hospitalisation stay prior to positive test was 5 days for cases (Table 1).

**Table 1.** Characteristics of the study population

Characteristics	Cases, *N* = 192 (%)	Controls, *N* = 192 (%)
Female sex, *N* (%)	99 (51·6)	99 (51·6)
Age, years		
Mean ± s.d.	79·8 ± 11·0	79·6 ± 10·7
Median	83·0	83·0
Min–Max	40·0–96·0	41·0–96·0
Hospital, *N* (%)		
Örebro University Hospital	107 (55·7)	107 (55·7)
Karlskoga Hospital	63 (32·8)	63 (32·8)
Lindesberg Hospital	22 (11·5)	22 (11·5)
Type of ward, *N* (%)		
Internal medicine/geriatrics	150 (78·1)	150 (78·1)
Surgery	12 (6·3)	12 (6·3)
Infectious diseases	19 (9·9)	19 (9·9)
Other	11 (5·7)	11 (5·7)
Hospitalisation time, days		
Mean value ± s.d.	16·7 ± 14·1	10·9 ± 10·3
Median	12·0	7·0
Min–Max	2·0–82·0	2·0–52·0
Hospitalisation time prior to positive test, days		
Mean value ± s.d.	8·0 ± 8·4	–
Median	5·0	–
Min–Max	1·0–59·0	–

**Fig. 1.**
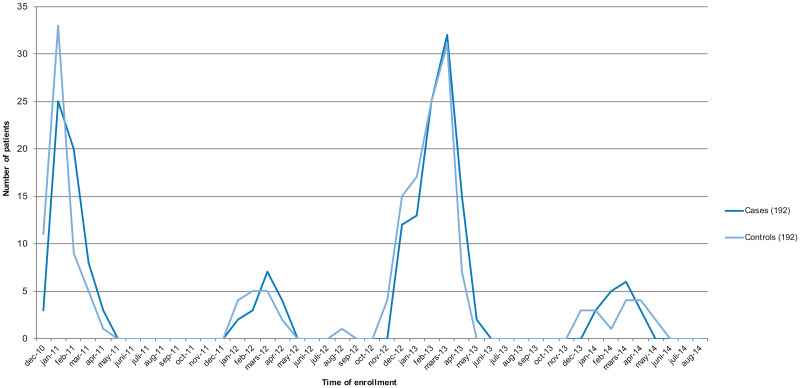
Number of cases and controls enrolled per month during the study period. The figure shows a biennial pattern of norovirus epidemic spread.

Cases and controls were matched with respect to time of admission, and were admitted during epidemic norovirus years (Fig. 1). Most of the cases occurred in 2010–2011 and 2012–2013, which is consistent with the Public Health Agency of Sweden national reports of viral gastroenteritis. The disease, which has a pattern of reaching epidemic levels every 2 years, was more frequent in 2010–2011 and 2012–2013 in Sweden. Details can be found in the PAS database [15].

**Fig. 2.**
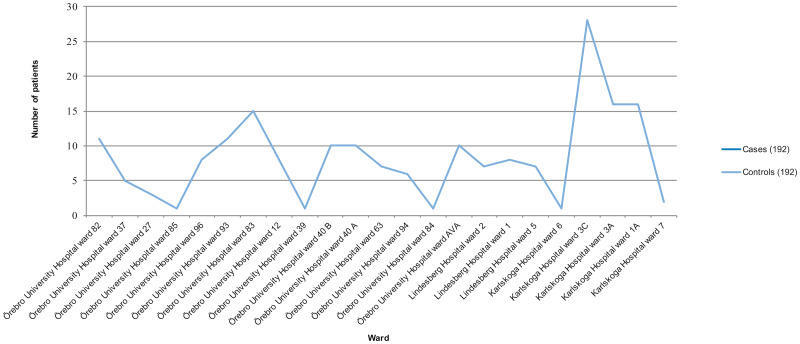
Number of cases and controls in each ward. The curve of cases is hidden behind the curve of controls, since controls were enrolled at the same time as cases due to matching. EW, emergency ward.

All case–control pairs were treated in the same ward (Fig. 2). The number of cases was generally higher in wards at Karlskoga Hospital compared with the other two hospitals.

### Use of PPIs

In 45 pairs the case, but not the control, was exposed (i.e. used PPIs), while in 26 pairs the control, but not the case, was exposed. There was a significantly increased risk of contracting norovirus infection in patients on PPI therapy, compared with patients not on PPIs (OR 1·73, 95% CI 1·07–2·81; *P* = 0·02).

## DISCUSSION

This study is, to the best of our knowledge, the first to investigate the relationship between PPI therapy and norovirus infection. In-patients affected by norovirus infection genogroup II were to a greater extent using PPI at admission to hospital. The interpretation of OR in a case–control study depends on the selection of controls [16]. In this study, they were selected based on the admission date of the cases, so-called ‘risk-set sampling’. This means that the OR (1·73) could be compared with the incidence ratio in cohort studies, a type of relative risk [16]. Our results suggest that a patient using PPI has an increased risk of contracting norovirus, compared with a patient in the same ward who is not on PPI therapy.

The patients’ gender, age, date of admission and ward were similar among the cases and controls, due to matching. This means that the risk of confounding within these factors is low. Through all seasons, the number of cases was high at Karlskoga Hospital, which ought to be investigated further regarding possible need for hygiene interventions.

The descriptive statistics demonstrate that the cases had a mean hospital stay of 17 days, and the controls of 11 days. No adjustments have been carried out for this discrepancy. Patients who have a norovirus infection most likely have a prolonged treatment period due to this acute disease as well as to exhaustion and dehydration during recovery. The course of the disease in healthy people is often short-term, 12–60 h. Elderly people and children may suffer a more protracted disease course, of up to 6 weeks [1, 2, 13]. The median hospitalisation time prior to positive test was 5 days for cases, and the median hospitalisation time was 12 days for cases and 7 days for control patients. It is possible that all of the extended period in hospital for cases was caused by the infection. However, this study did not establish whether the extended hospital stay of the cases was due to norovirus infection, and it cannot be excluded that extended hospital stays caused by other factors had an impact on the results.

Possible reasons why PPIs increase the risk of norovirus infection may be that they inhibit gastric acid, affect the intestinal flora or interfere with the immune system. Gastric acid is an important protection against microbes [3]. Therefore, PPI therapy may lead to greater survival of undesirable microorganisms.

Normally the gut flora has a protective function against pathogenic microbes and activates the immune system. PPIs disrupt the intestinal flora by causing overgrowth of bacteria, mainly in the colon, but also in the small intestine where bacteria normally do not exist [3]. This may be due to bacteria having higher survival in the more alkaline conditions of the ventricle [3]. Furthermore, PPIs probably inhibit P-glycoprotein in the epithelium of the GI tract, a protein that pumps back contaminants to the GI tract. Lack of P-glycoprotein probably helps bacteria to attach to the mucosa of the GI tract; thus PPIs may facilitate for microbes to infect the host [5].

Many cells in the body, such as erythrocytes and intestinal epithelial cells, express histo-blood group antigens (HBGAs) on the surface of the cell, as well as certain bacteria in the intestinal flora [2]. Norovirus could bind to HBGAs to infect B-lymphocytes and the virus may use HBGA to pass through the epithelium [2]. Intestinal bacteria expressing HBGA may facilitate norovirus infection by acting as co-factors during norovirus attacks on B-lymphocytes [17]. Furthermore, it has been demonstrated that reduction of the intestinal flora caused by antibiotics can lead to decreased titres of norovirus in mice [17]. Therefore, increased gut flora in the GI tract presumably create facilitating conditions for norovirus infection.

It has been reported that normal doses of PPI in patients with GORD reduce the number of T-lymphocytes in the oesophageal epithelium [9]. Since PPI affects T-lymphocytes, this may be one reason why the risk of norovirus infection increases during PPI therapy.

PPIs have previously been considered to be drugs without serious side effects but new findings suggest that PPIs may increase the risk of various conditions, such as *C. difficile* infection, pneumonia and fractures [4, 13, 19]. Jena *et al*. report that PPIs, in addition to increasing the risk of pneumonia, also increase the risk for a range of other medical conditions, such as chest pain, deep vein thrombosis and rheumatoid arthritis [20]. They propose that the relationship between pneumonia and PPIs is caused by an unknown confounder. Risk of confounding is proposed to be higher if the current exposure is linked to many different outcomes [20]. Consequently, the results of this study may be due to an unknown confounder.

The present study is, to the best of our knowledge, the first study examining the connection between PPI use and norovirus infection, and the findings should be investigated further. The results motivate more comprehensive studies.

The study has limitations. Controls were not tested for norovirus to confirm lack of asymptomatic infection which may lead to misclassification bias. Furthermore, the retrospective information regarding PPI therapy in the admission records may in some cases be incorrect. For instance, PPI treatment may have been altered within the hospitalisation period, which this study did not adjust for. Since the study only included patients whose hospital admission records included current medicines, a greater number of patients admitted without this type of documentation may have had more extensive use of medication. However, these factors regarding exposure may affect both cases and controls, and are therefore non-differential misclassifications. Despite these potential misclassifications a significant result is presented.

The study does not account for other diseases, immunosuppression, or other medicines such as antibiotics – factors that might have influenced both exposure and outcome, which may therefore be confounders. Nor does the study take into account whether, during hospitalisation, cases were affected by gastroenteritis caused by viruses other than norovirus. None of the controls had any kind of symptomatic gastroenteritis during the hospitalisation, which may result in selection bias. It is possible that some of the cases had gastroenteritis other than norovirus during hospitalisation, such as *C. difficile*, which may have affected the results.

A percentage of the population are immune to norovirus, which has not been verified in this study. As no adjustments have been carried out, this could have had an impact on the results. There is a risk that the real cause of the increased incidence of norovirus infection was not PPI drugs, but the condition being treated, since no adjustments have been made. Therefore, all these potential confounders should be considered in future studies.

In order to increase the external validity, future studies should cover a larger region than Örebro County and include a larger number of participants. This is a study of in-patients; studies of out-patients should be performed to investigate whether the relationship also exists in non-institutional care. Prospective cohort studies should be considered for higher precision and internal validity of exposure and outcome. Stratification for the PPI dose should be performed in the statistical analysis to examine whether dosage affects the result. Furthermore, future studies should include other genogroups of norovirus in order to provide a complete picture of the association between PPI and norovirus infection.

Preclinical studies in animals, for example rodents or pigs, should be performed before randomised controlled trials are performed in humans. Studies in rodents have been performed with murine norovirus [21]. Comparing surrogates with human norovirus is complicated, as these viruses may differ in pathogenesis even though they are similar in structure [2]. For example, murine norovirus infections show no symptoms in mice with an intact immune system, unlike norovirus infection in healthy people [18]. Human norovirus is capable of infecting pigs. However, pig studies are more expensive than rat studies [21]. Since no studies have been accomplished where calicivirus is exposed to pH <2 [11], such research should be carried out to investigate the stability of the virus.

More epidemiological and mechanistic studies should be done before any intervention studies are carried out.

Proton pump-inhibiting drugs have previously been shown to increase the risk of other diseases and many patients use them without indication [22, 23]. Omeprazole was the sixth most prescribed drug in the US in 2011 [4], and there is a global discussion about whether these substances are being overprescribed. An American study from 2010 reports that in 36·1% of cases (*n* = 946), no valid indication of the drug was found and almost half of the patients with ongoing PPI treatment were not followed [22]. At a hospital in Singapore, Akram *et al*. [23] demonstrated that >80% of the patients on PPI therapy received the drug without valid indication. Some patients continue to use the substances after an initial treatment period, due to rebound phenomena [24]. The results of this study contribute to the debate about whether there should be a more moderate approach to PPIs. Health professionals and patients should receive more information about PPI risks, and at-risk patients with a weak indication for PPI therapy should be considered to discontinue the medicine. It can also be questioned whether it is ethical to sell PPIs over the counter or market them using public media.

## CONCLUSIONS

The results of our study show that PPIs appear to be a risk factor for norovirus infection. The study design does not exclude confounders, which may have affected the outcome. The results are, however, of interest as no previous studies have been done to establish this relationship. PPIs appear to be a risk factor for norovirus infection, and our results motivate future studies to further examine this association.
